# Supplementary material for: Preclinical assessment of checkpoint blockade combined with DNA methyltransferase inhibition in high-risk pediatric brain tumors reveals limited therapeutic synergy
Source: Neurooncol Adv. 2025 Nov 13;8(1):vdaf241. doi: 10.1093/noajnl/vdaf241 (PMC12803780; doi:10.1093/noajnl/vdaf241)
Supplement: vdaf241_Supplementary_Data [file vdaf241_supplementary_data.docx]

**SUPPLEMENTAL MATERIALS**

**Cell Lines:**

| **Human Cell Lines** | **Tumor Type** | **Source** |
| --- | --- | --- |
| PBT-14FHTC | DMG, H3.3K27M | Olson^36^ |
| PBT-22FHTC | DMG, H3.3K27M | Olson^36^ |
| PBT-29FHTC | DMG, H3.3K27M | Olson^36^ |
| SU-DIPG-XIII | DMG, H3.3K27M | Monje^37^ |
| SU-DIPG-XVII | DMG, H3.3K27M | Monje^37^ |
| PBT-27FHTC | DMG, H3.1K27M | Olson^36^ |
| SU-DIPG-IV | DMG, H3.1K27M | Monje^37^ |
| CCHMC-DIPG-1 | DIPG, H3WT | Drissi^38^ |
| PBT-24FHTC | DIPG, H3WT | Olson^36^ |
| VUMC-DIPG-10 | DIPG, H3WT | Hulleman |
| Med-114FHTC | Medulloblastoma, Group 3, *MYC* amp | Olson^39^ |
| Med-411FHTC | Medulloblastoma, Group 3, *MYC* amp | Olson^39^ |
| Med-2112FHTC | Medulloblastoma, Group 3, *MYC* amp | Olson^39^ |
| Med-813FHTC | Medulloblastoma, Group 3, *MYC* amp | Olson^39^ |
| ATRT-311FHTC | ATRT, SHH | Olson^39^ |
| ATRT-310FHTC | ATRT, SHH | Olson^39^ |
| CHLA-04 | ATRT, SHH | ATCC |
| BT-12 | ATRT, MYC | Children’s Oncology Group^40^ |
| BT-16 | ATRT, MYC | CHOP^41^ |
| CHLA-06 | ATRT, MYC | ATCC |
| PBT-05FHTC | HGG, *MYC-N* amp | Olson^39^ |
| GBM-110FHTC | HGG | Olson^39^ |
| GBM-511FHTC | HGG | Olson^39^ |
| SU-pcGBM2 | HGG | Monje^37^ |
| EPD-210FHTC | Ependymoma | Olson^39^ |

| **Murine Cell Lines** | **Tumor Type** | **Source** |
| --- | --- | --- |
| IUE-24-C5 | Pdgfra^D842V^, dominant neg *Trp53*, H3 WT | Phoenix^42^ |
| IUE-K27M-APP | H3.3K27M, *Trp53-Atrx*-KD, PDGFRA-overexpression | Salomoni^34^ |
| IUE-24-B1 | Pdgfra^D842V^, dominant neg *Trp53*, H3.3K27M | Phoenix, Hulleman^32^ |
| IUE-24-B7 | Pdgfra^D842V^, dominant neg *Trp53*, H3.3K27M | Phoenix, Hulleman^32^ |
| SHH57835 | *Ptch1^+/-^,* *Trp53* KO | Roussel^33^ |
| 7444 | *c-Myc* CRISPR, *Trp53* loss, *Cdkn2c* loss | Roussel^35^ |

**qPCR Primers:**

| **Gene Target** | **Taqman Assay** |
| --- | --- |
| *CTAG1B (NY-ESO-1)* | Hs00265824_m1 |
| *MAGEA1* | Hs00607097_m1 |
| *ACTB* | Hs99999903_m1 |
| *PrameL1* | Mm00473193_m1 |
| *Trap1a* | Mm00495785_m1 |
| *MageA4* | Mm00522322_s1 |
| *Rhox5* | Mm00476718_m1 |
| *Irf7* | Mm00516788_m1 |
| *GusB* | Mm01197698_m1 |

**Antibodies:**

| **Target** | **Source** | **Application** |
| --- | --- | --- |
| 5-methylcytosine | Sigma Aldrich (MABE146) | Dot Blot |
| PE Mouse IgG1, κ Isotype control | BioLegend (400114) | Flow |
| PE Mouse IgG2a, κ Isotype control | BioLegend (400214) | Flow |
| APC Mouse IgG2a, κ Isotype control | BioLegend (400220) | Flow |
| APC Rat IgG2a, κ Isotype control | BioLegend (400512) | Flow |
| PE anti-human HLA-A,B,C | BioLegend (311406) | Flow |
| APC anti-human HLA-A,B,C | BioLegend (311410) | Flow |
| PE anti-human CD274 (B7-H1, PD-L1) | BioLegend (329706) | Flow |
| APC anti-human CD274 (B7-H1, PD-L1) | BioLegend (329708) | Flow |
| APC anti-mouse H-2K^b^/H-2D^b^ | BioLegend (114614) | Flow |
| PE anti-mouse CD274 (B7-H1, PD-L1) | BioLegend (124308) | Flow |
| CD3 | Roche (790-4341),  Abcam (ab11089, ab16669) | IHC |
| CD4 | Abcam (ab183685) | IHC |
| CD8 | Cell Signaling (98941),  Abcam (ab209775) | IHC |
| F4/80 | Abcam (ab6640) | IHC |
| PD-1 | Abcam (ab214421) | IHC |
| FoxP3 | ThermoFisher (14-5773-82) | IHC |

**CyTOF Antibodies:**

| **Antibody** | **Metal** | **Cell Types/Function** |
| --- | --- | --- |
| CD45 | 89Y | Leukocytes |
| MHCII (IAIE) | 113Cd | APC |
| CD8a | 116Cd | Cytotoxic T cells |
| CD44 | 141Pr | Memory |
| CD11b | 142Nd | Myeloid |
| IL-10 | 143Nd | Cytokine (anti) |
| CD103 | 144Nd | Tissue residency |
| CD4 | 145Nd | Helper T cells |
| CD11c | 146Nd | Dendritic Cells |
| CD223/LAG3 | 147Sm | Check point inhibitor |
| TNFα | 148Nd | Cytokine (pro) |
| PD-L1 | 149Sm | Check point inhibitor |
| Arginase 1 | 150Nd | Anti-inflammatory |
| Ly6C | 151Eu | Myeloid/monocytes |
| CD3 | 152Sm | T cells |
| CD16 | 153Eu | IgG receptor myeloid |
| CD284/TLR4 | 154Sm | Innate signaling |
| CD68 | 155Gd | Monocytes/macrophage |
| CD14 | 156Gd | Monocytes |
| Ly6G | 158Gd | Neutrophils |
| CD15 | 159Tb | Neutrophils |
| Ki-67 | 161Dy | Proliferation |
| Foxp3 | 162Dy | Tregs |
| TIGIT | 163Dy | Check point inhibitor |
| CD62L | 164Dy | Naïve |
| F4/80 | 165Ho | Macrophage |
| GATA-3 | 166Er | Transcription factor |
| CD127 | 167Er | IL7 R, ILCs T cells |
| CD64 | 168Er | Macrophage |
| CD206 | 169Tm | Anti-inflammatory Macrophage |
| RoRγt | 170Er | Transcription factor |
| PD-1 | 171Yb | Check point inhibitor |
| CD86 | 172Yb | Pro-inflammatory Macrophage |
| CD69 | 173Yb | Activation/residency |
| CCR2 | 174Yb | Recent infiltration |
| Tbet | 175Lu | Transcription factor |
| IL1β | 176Yb | Cytokine (pro) |

**­­**

**Supplemental FIGURES**

**Supplemental Figure 1:**


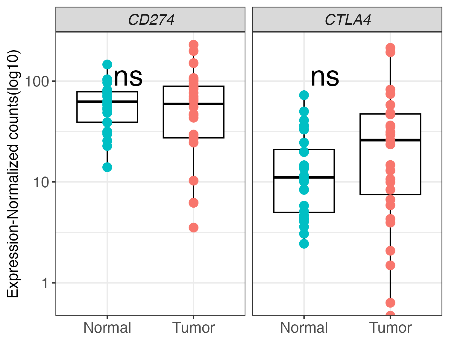

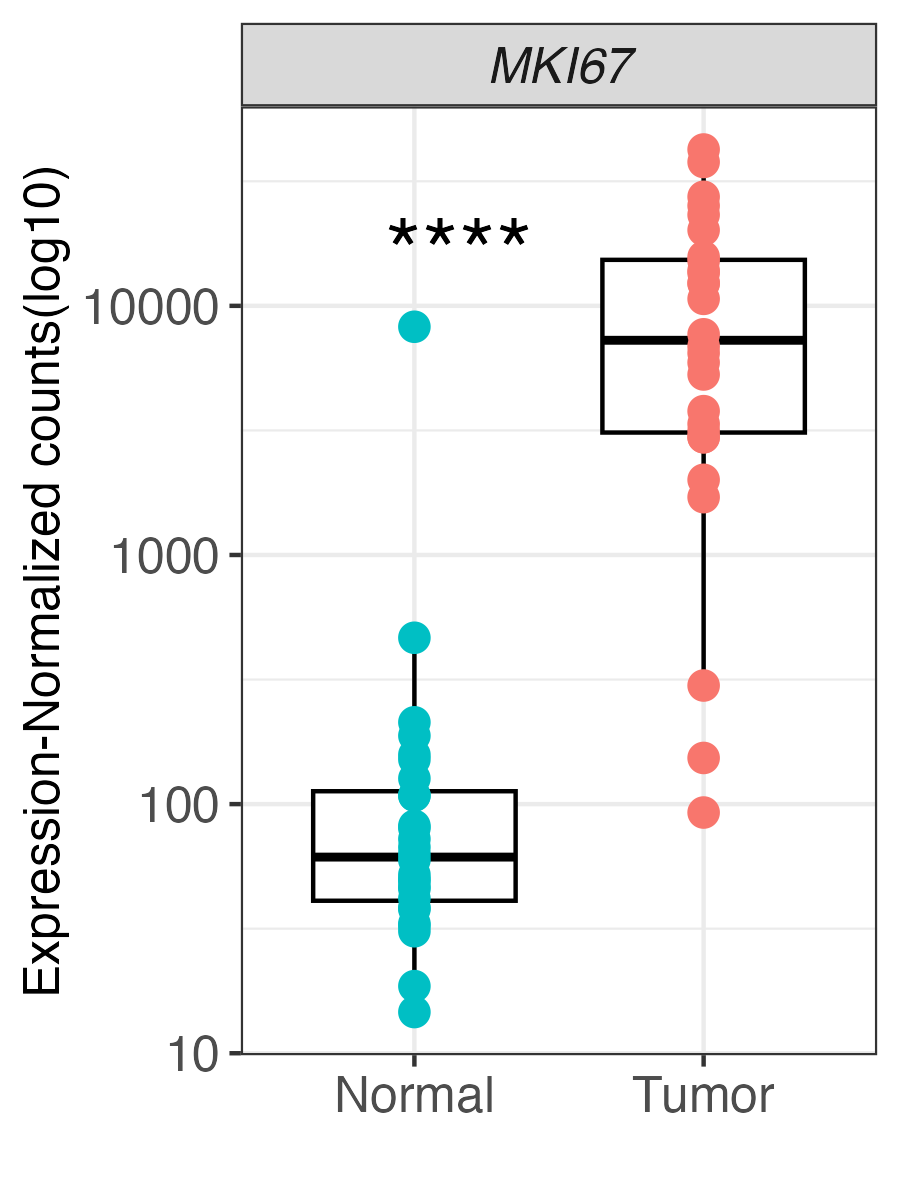

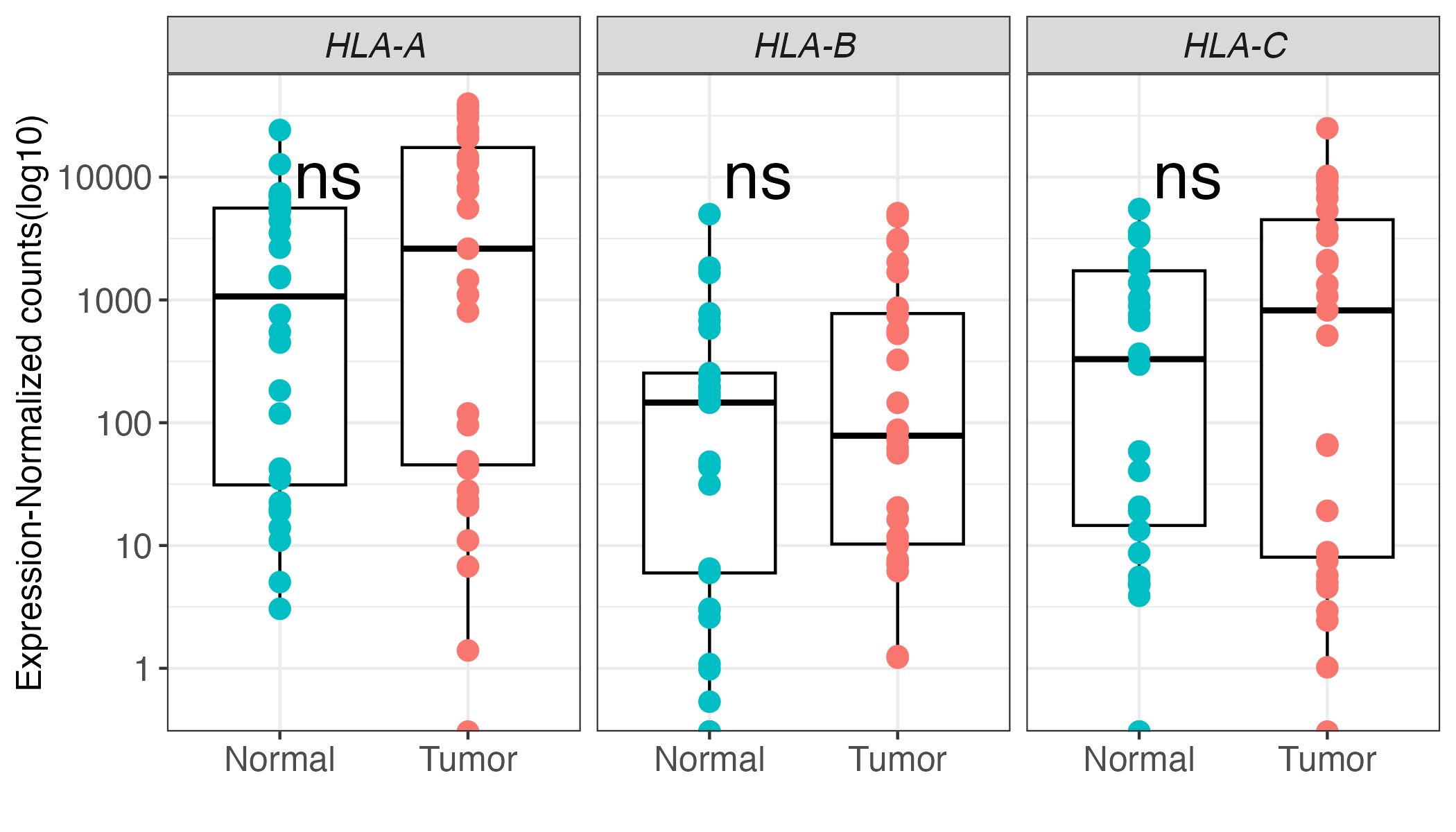

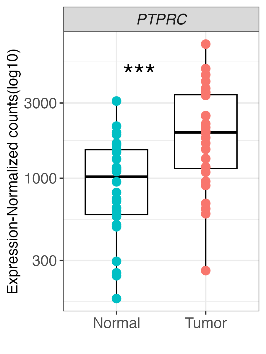

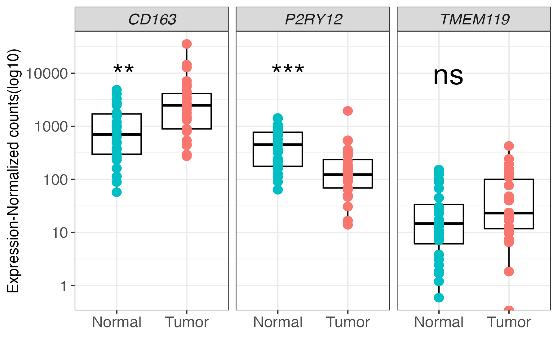

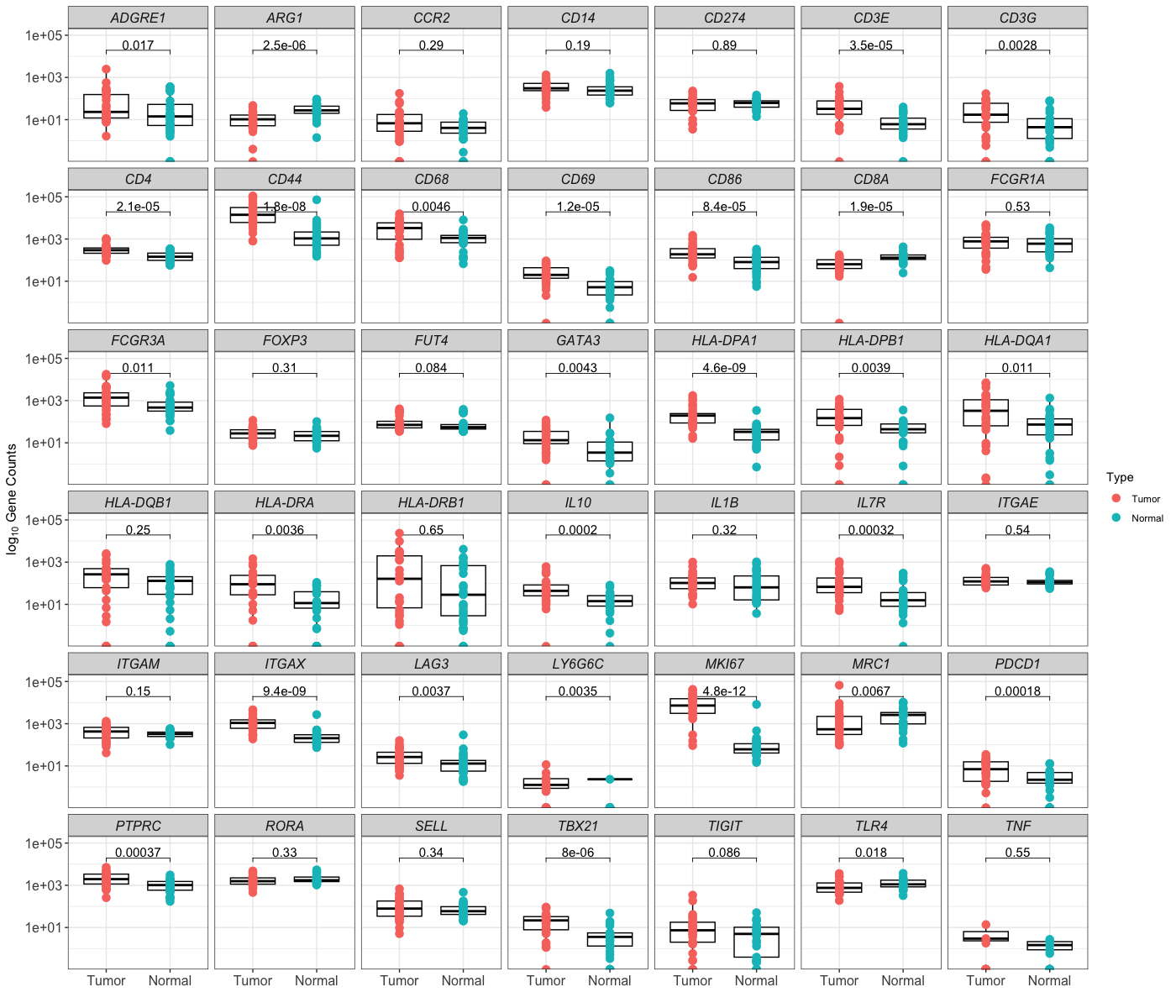

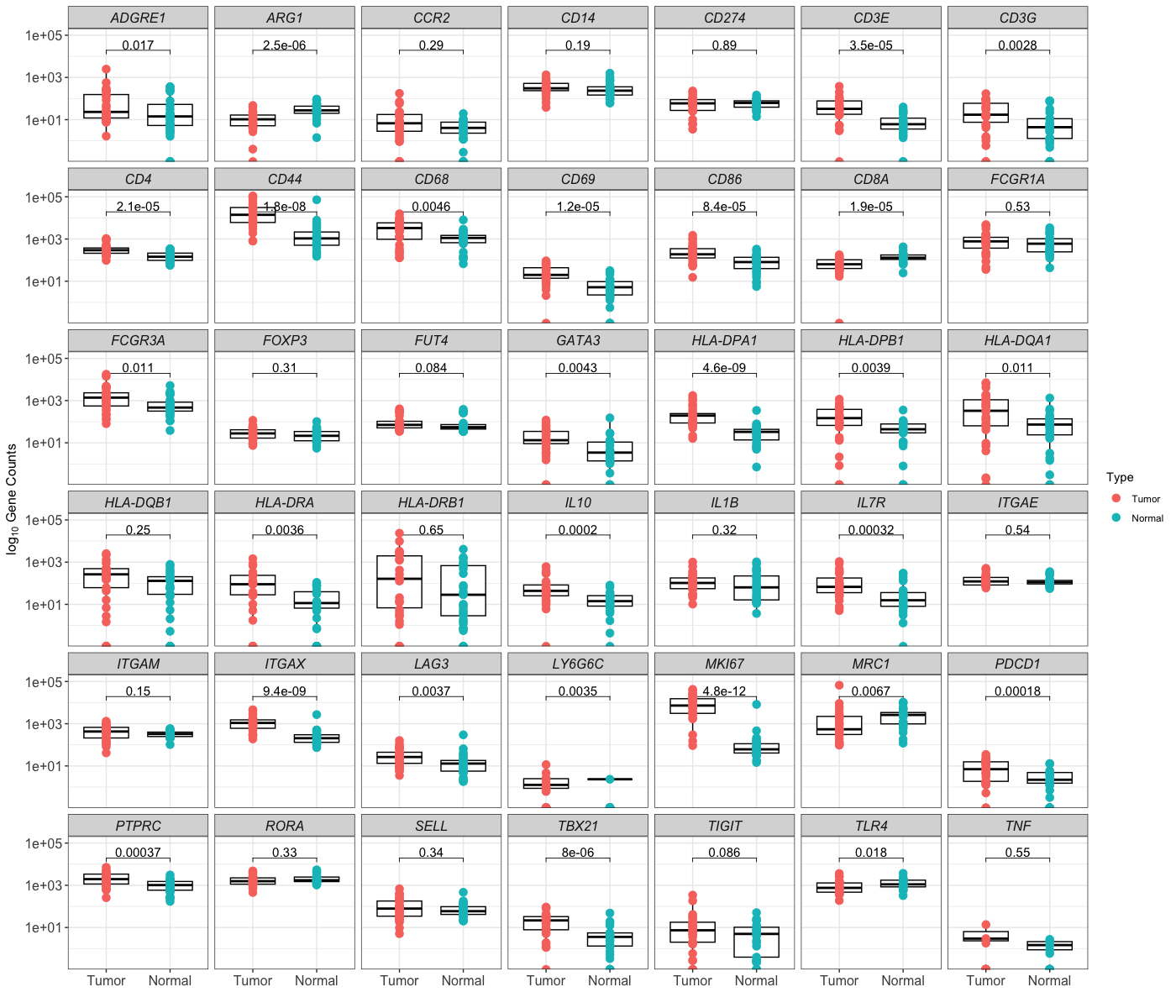

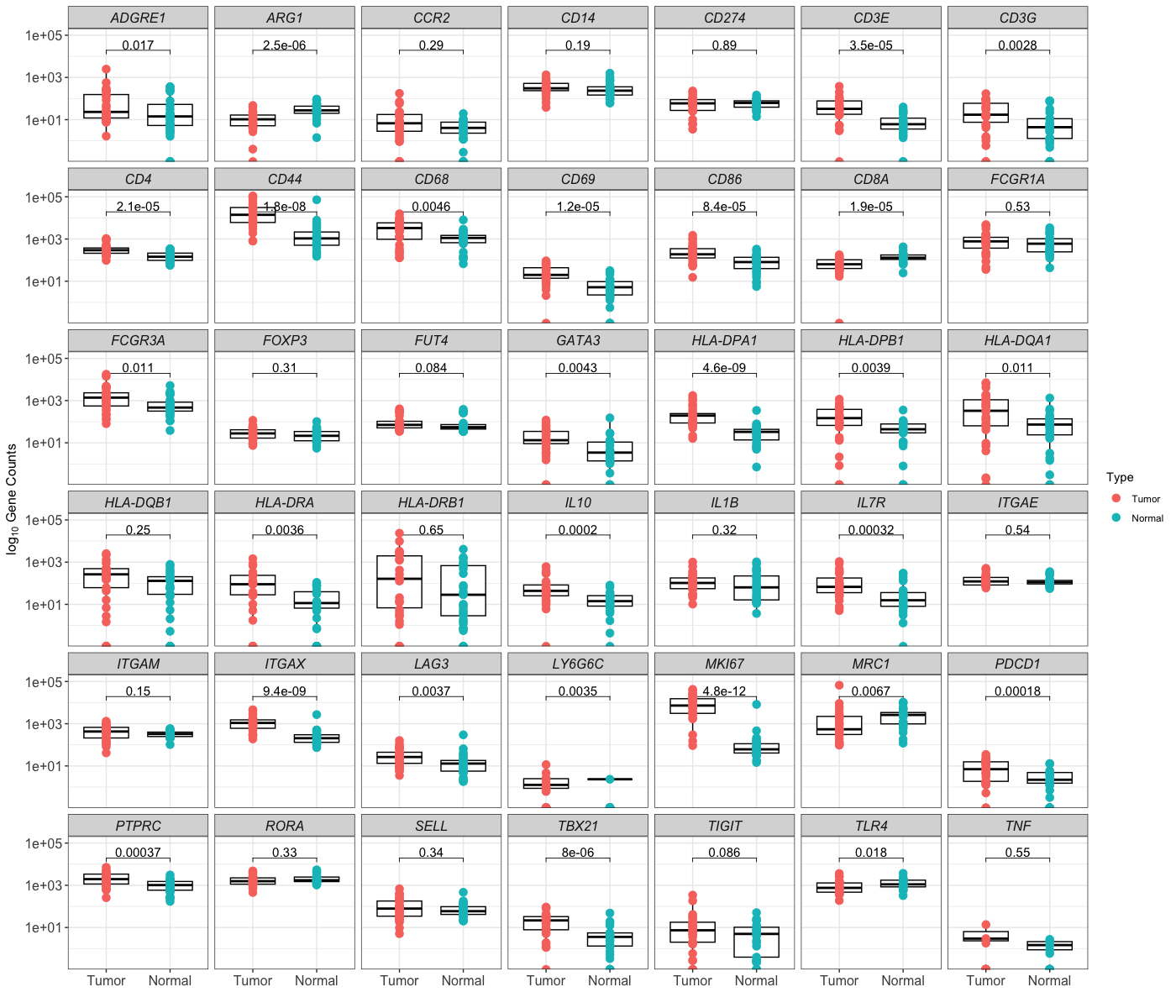

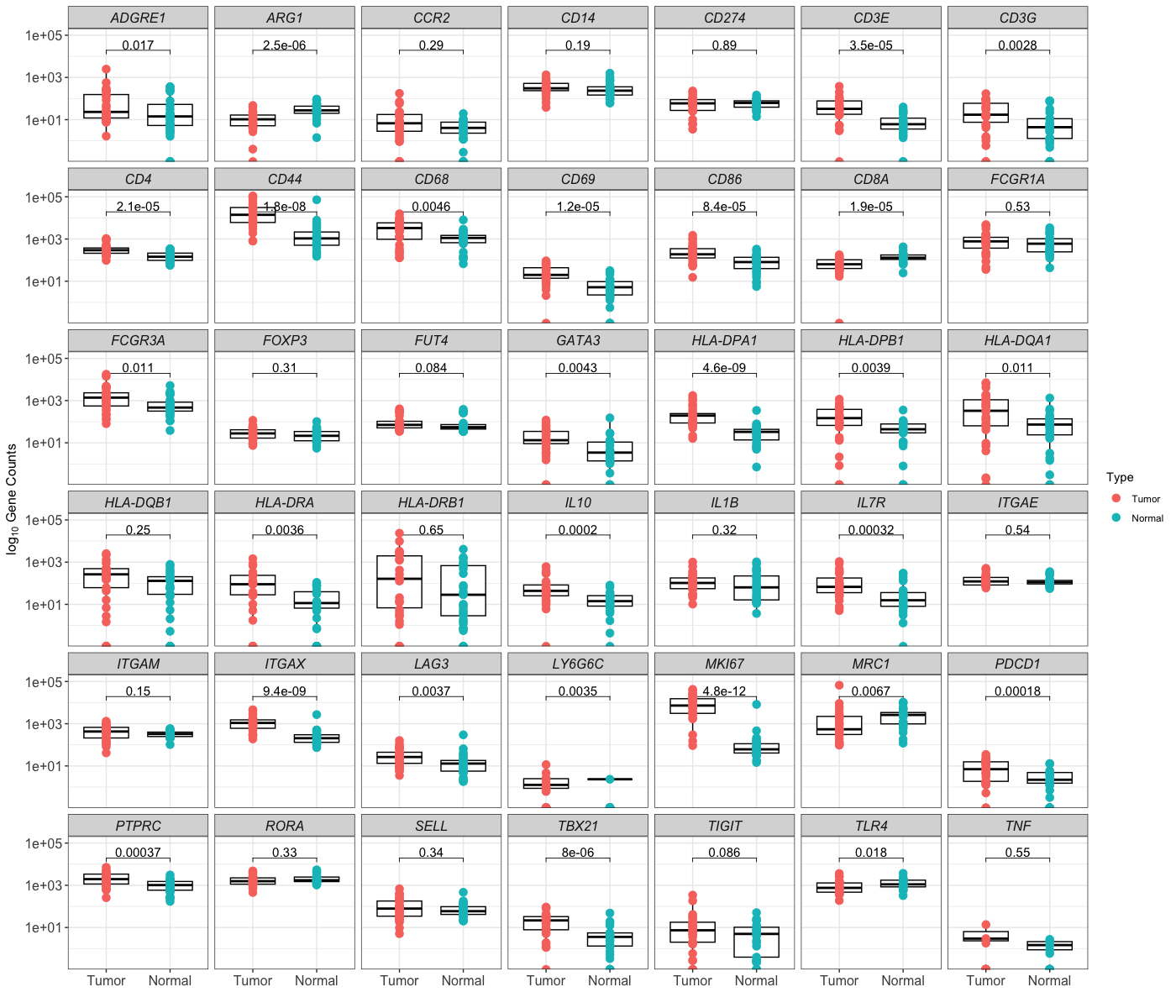


**S1. Select immune marker gene expression in DIPG/DMG tumor samples compared to normal brain.** Stars represent: * *P* ≤ 0.05, ** *P* ≤ 0.01, *** *P* ≤ 0.001, **** *P* ≤ 0.0001. ns = *P* > 0.05.

**Supplemental Figure 2:**


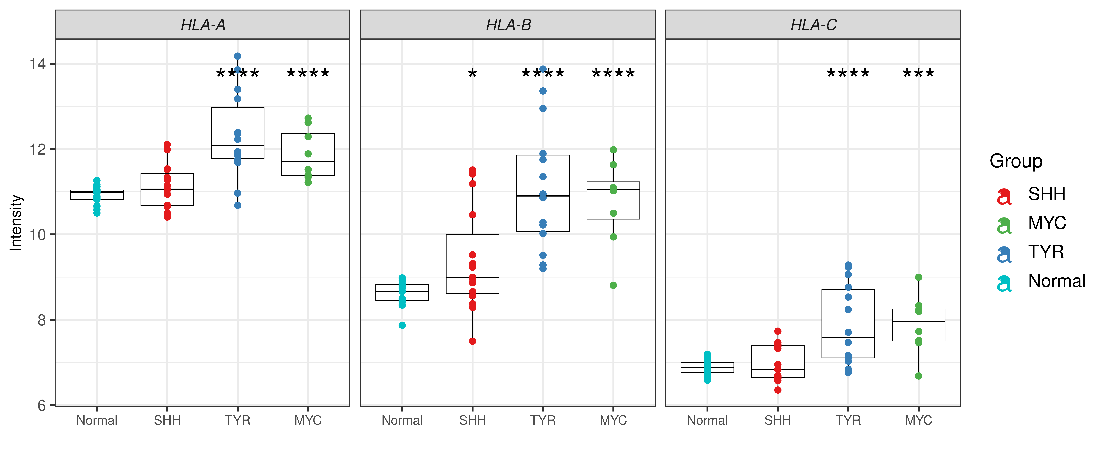

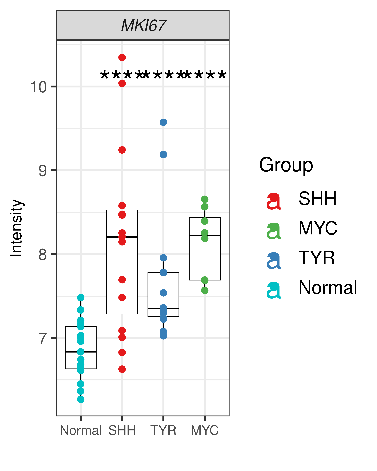

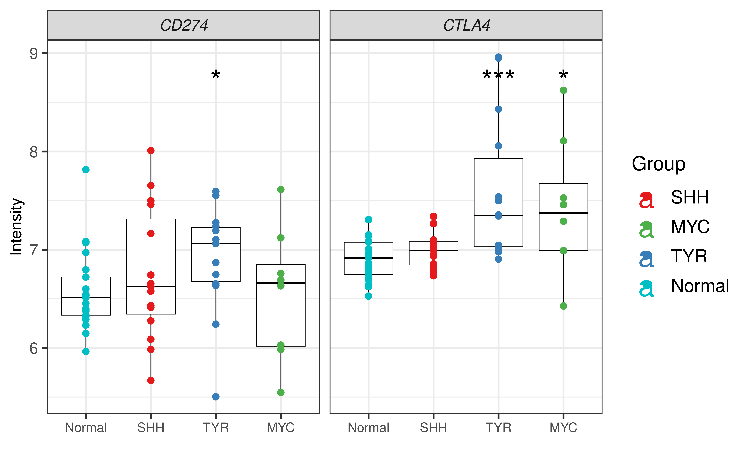

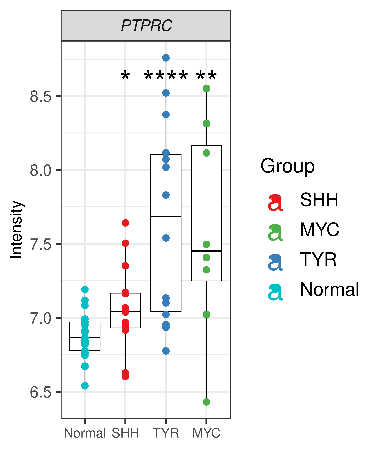

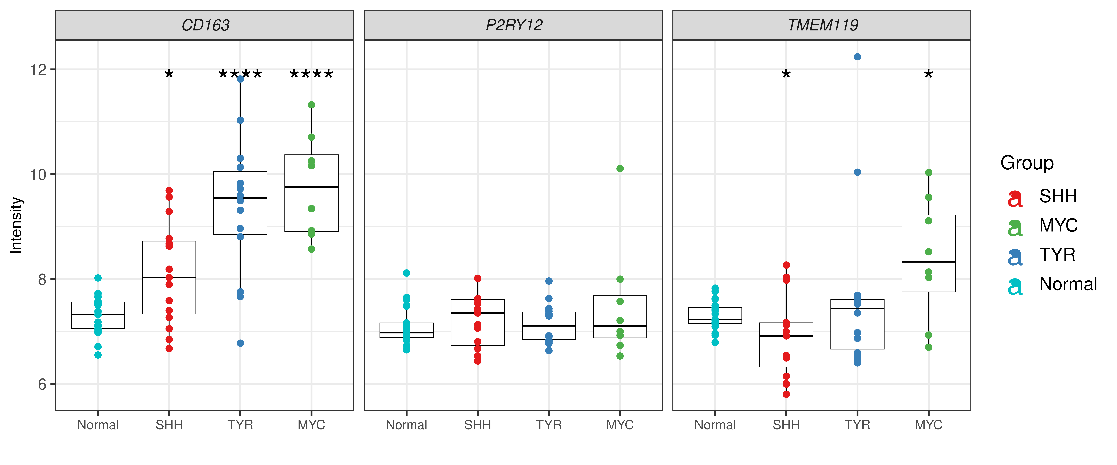


**­­­­**

**
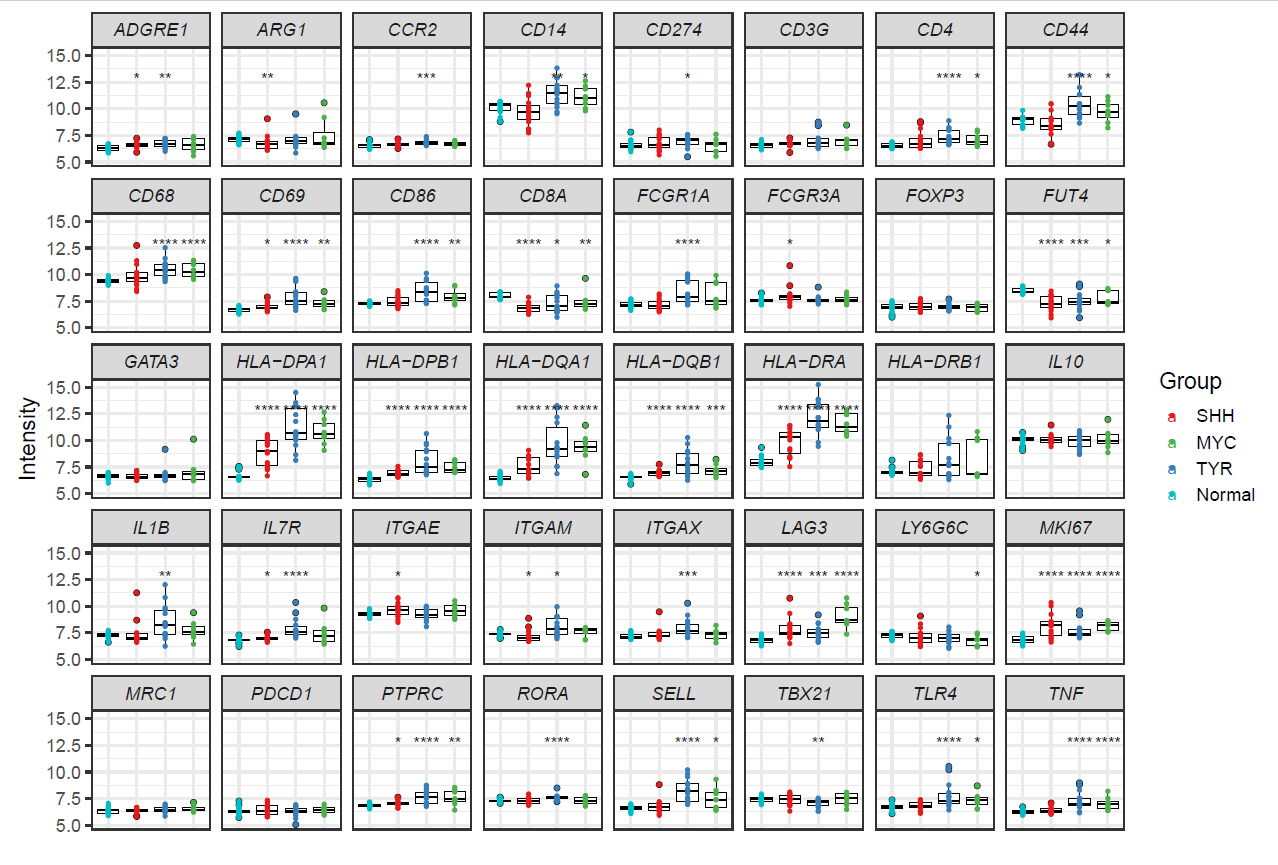
**

**S2. Select immune marker gene expression in ATRT tumor samples compared to normal brain.** Stars represent: * *P* ≤ 0.05, ** *P* ≤ 0.01, *** *P* ≤ 0.001, **** *P* ≤ 0.0001.

**Supplemental Figure 3:**


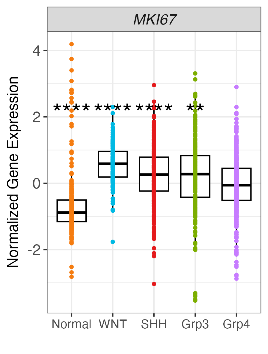

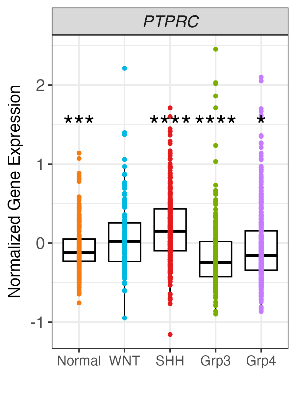

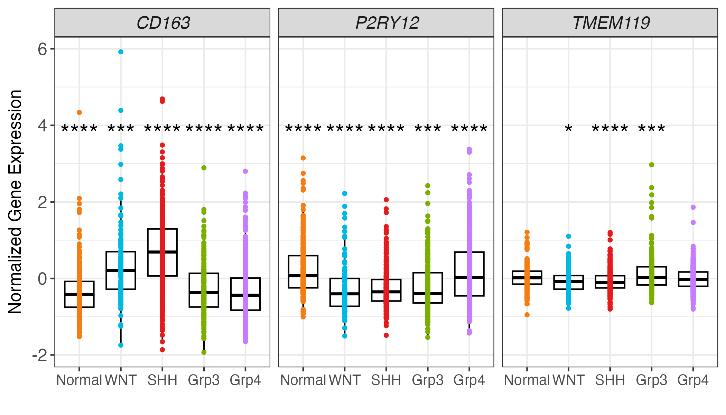

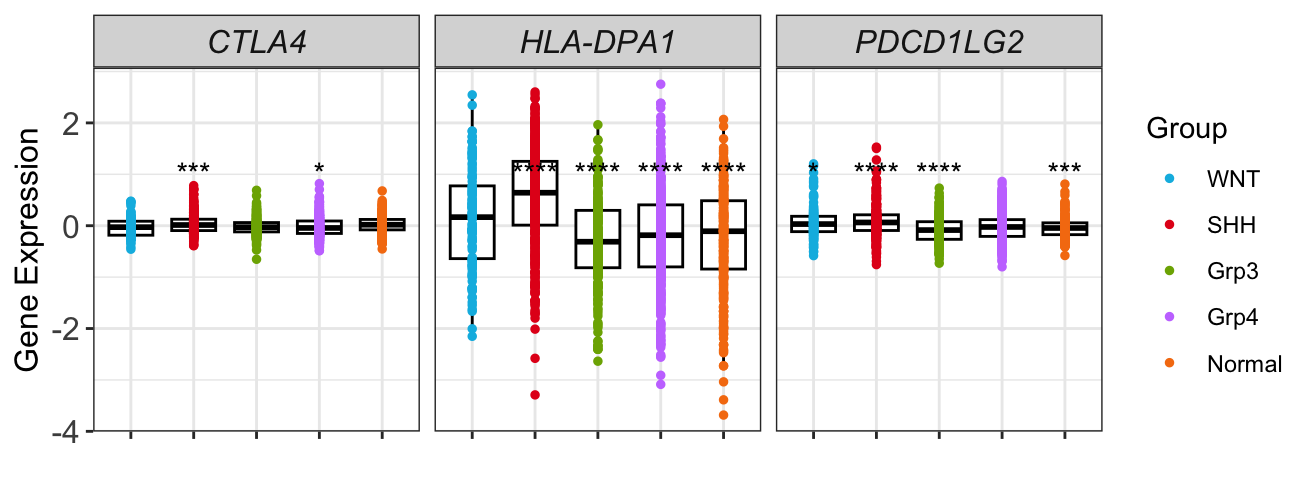

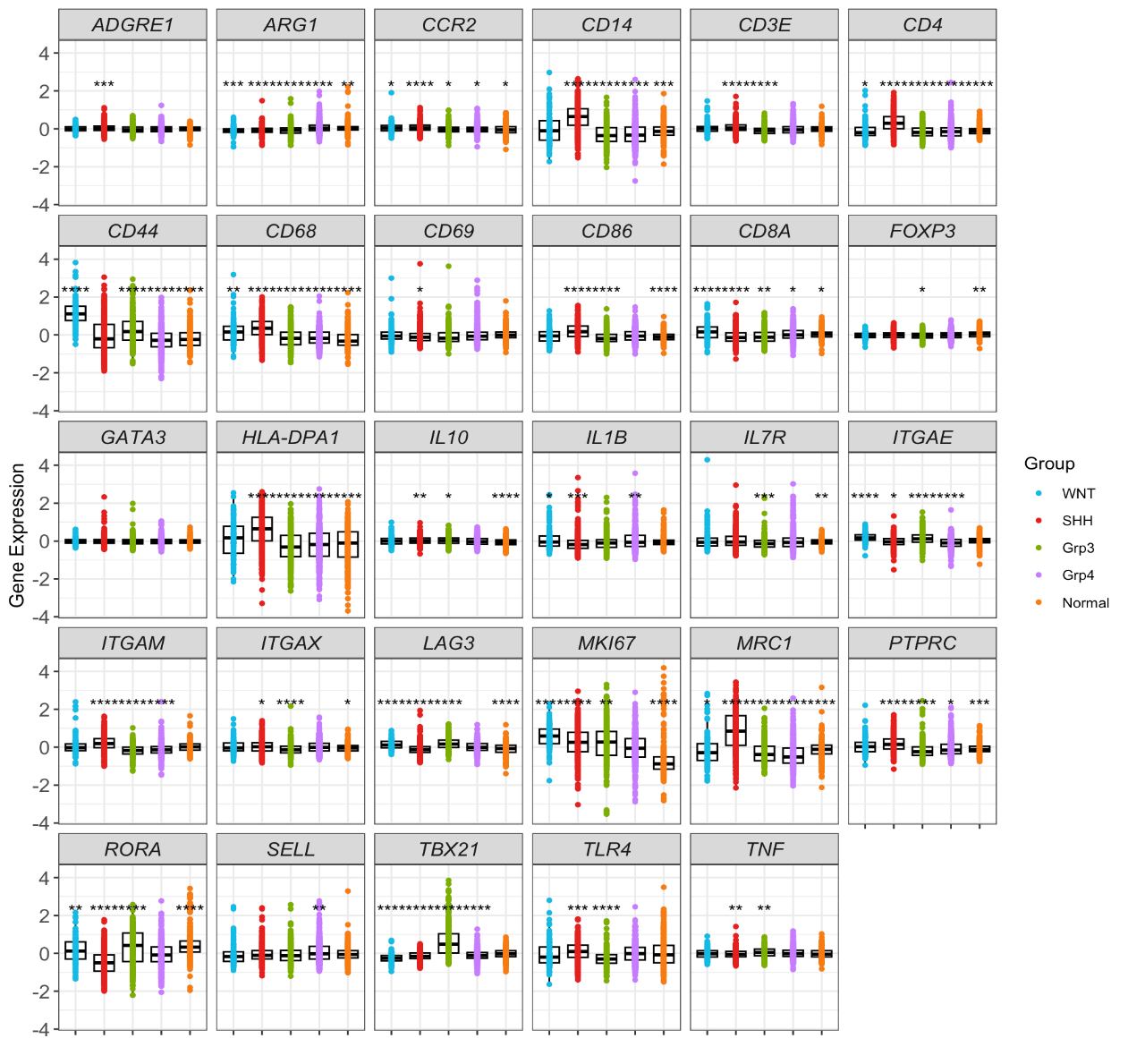


**­­­­­­­**

**S3. Select immune marker gene expression in medulloblastoma tumor samples compared to normal brain.** From Swartling data set (Weishaupt H, Johansson P, Sundstrom A, et al. Batch-normalization of cerebellar and medulloblastoma gene expression datasets utilizing empirically defined negative control genes. *Bioinformatics.* 2019; 35(18):3357-3364). Stars represent: * *P* ≤ 0.05, ** *P* ≤ 0.01, *** *P* ≤ 0.001, **** *P* ≤ 0.0001.

**Supplemental Figure 4:**


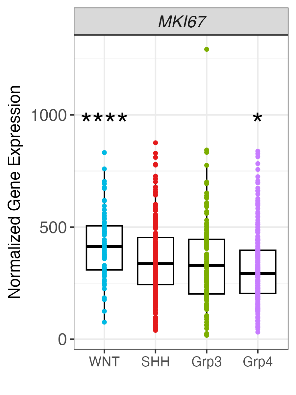

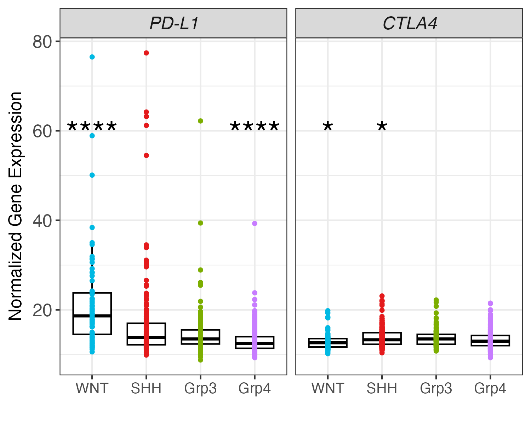

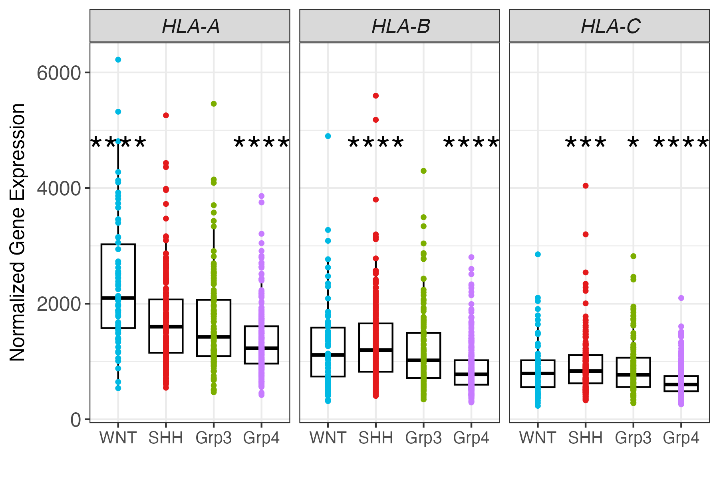

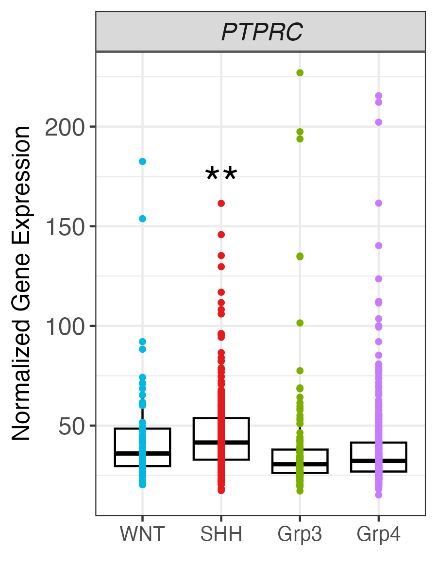

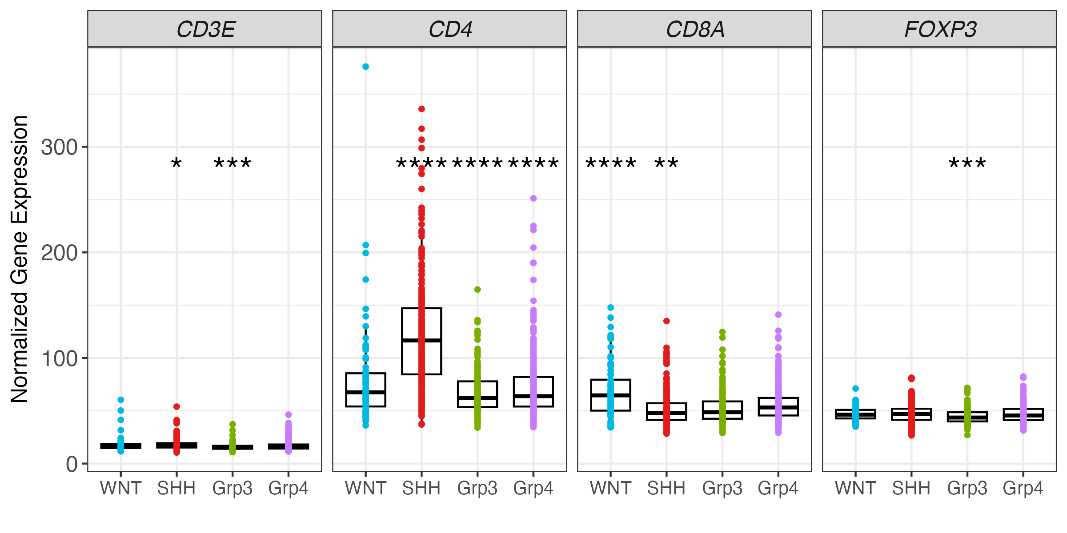

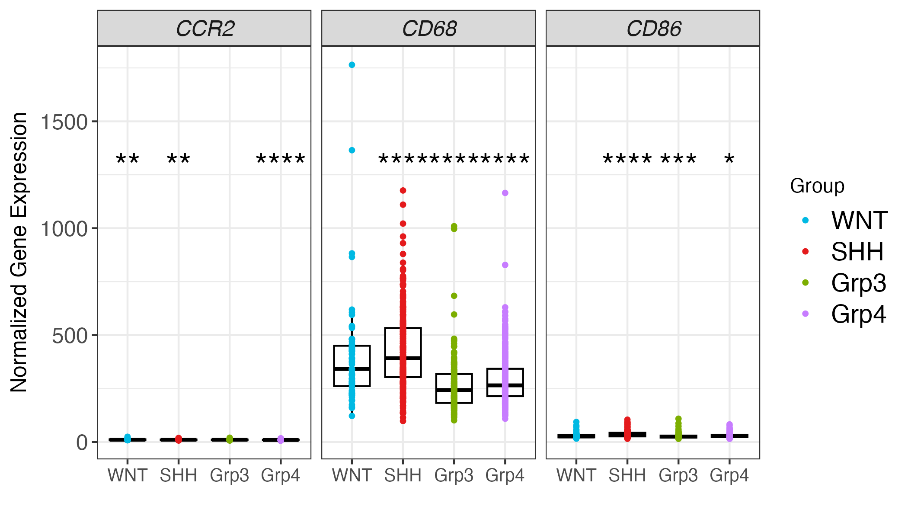

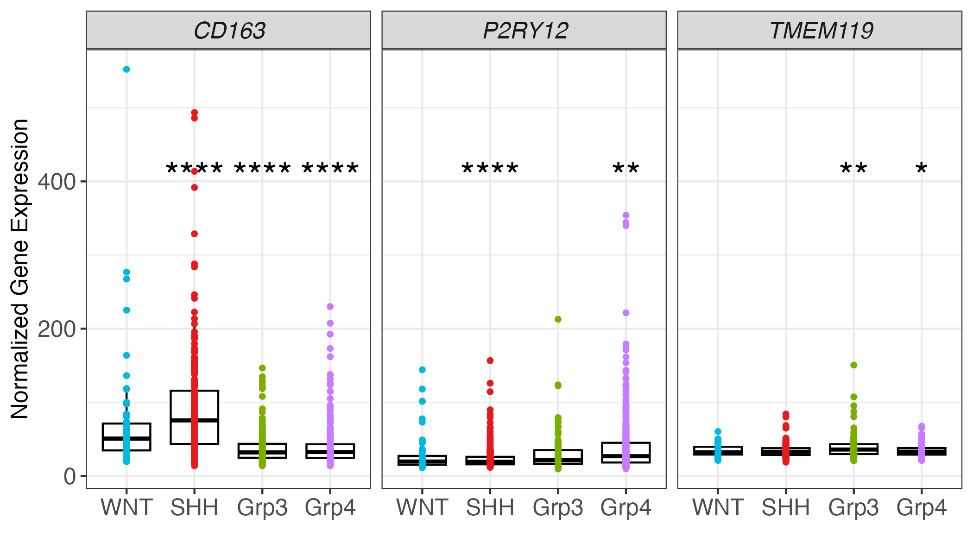


**S4. Select immune marker gene expression in medulloblastoma tumor samples.** From Cavalli data set (Cavalli FMG, Remke M, Rampasek L, et al. Intertumoral Heterogeneity within Medulloblastoma Subgroups. *Cancer Cell.* 2017; 31(6):737-754 e736). Data set includes 763 primary samples consisting of 70 WNT, 223 SHH, 144 group 3, and 326 group 4 samples. Stars represent: * *P* ≤ 0.05, ** *P* ≤ 0.01, *** *P* ≤ 0.001, **** *P* ≤ 0.0001.

**Supplemental Figure 5:**


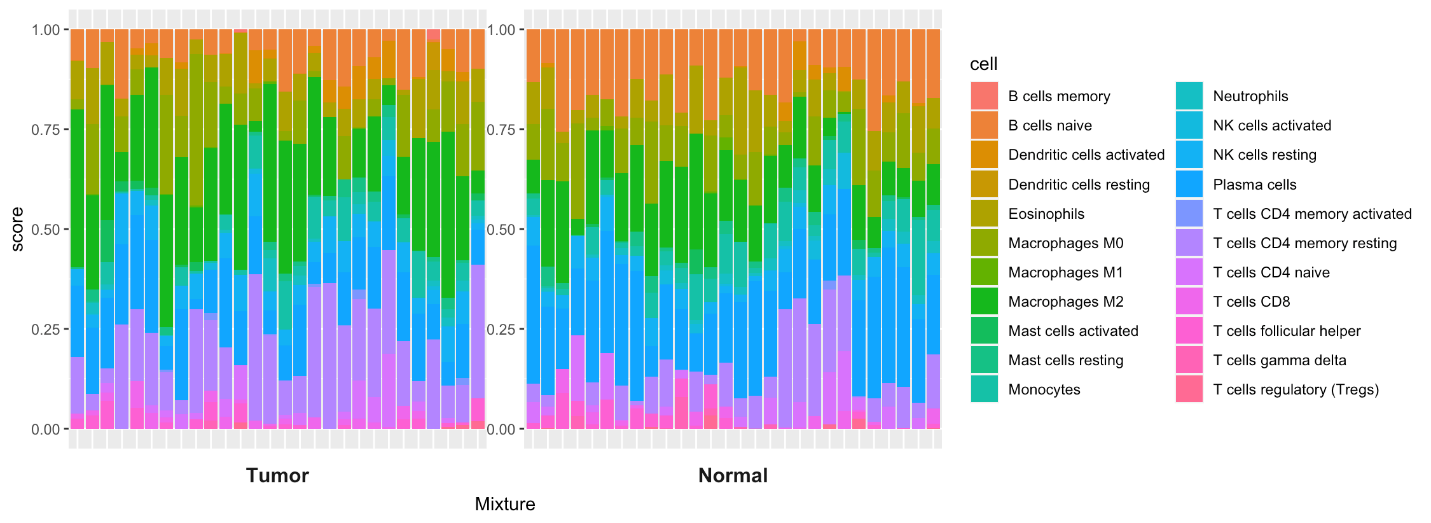
**A.**


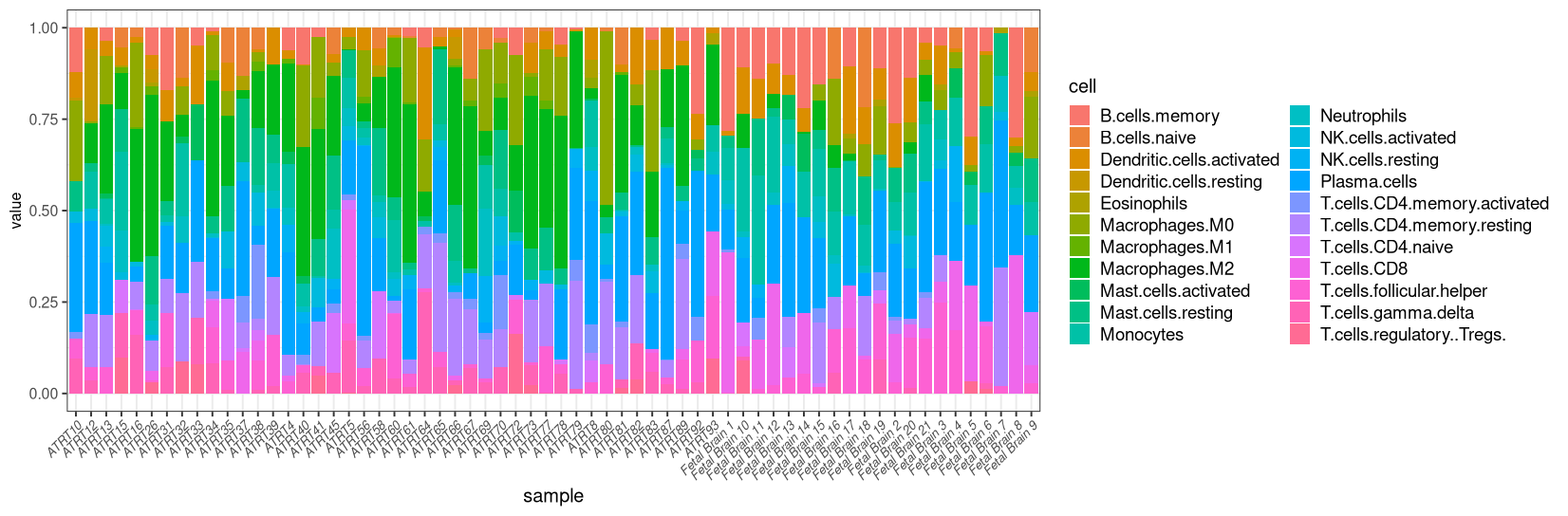
**B.**


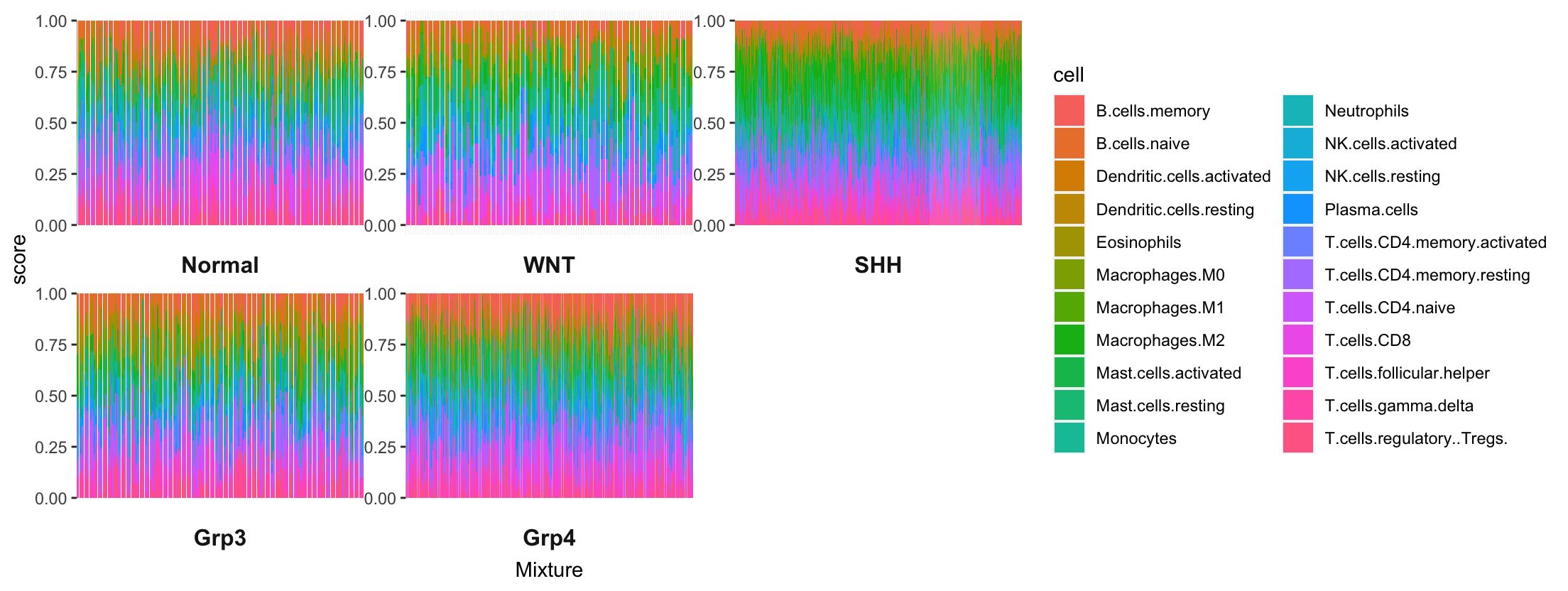
**C.**

**D.**
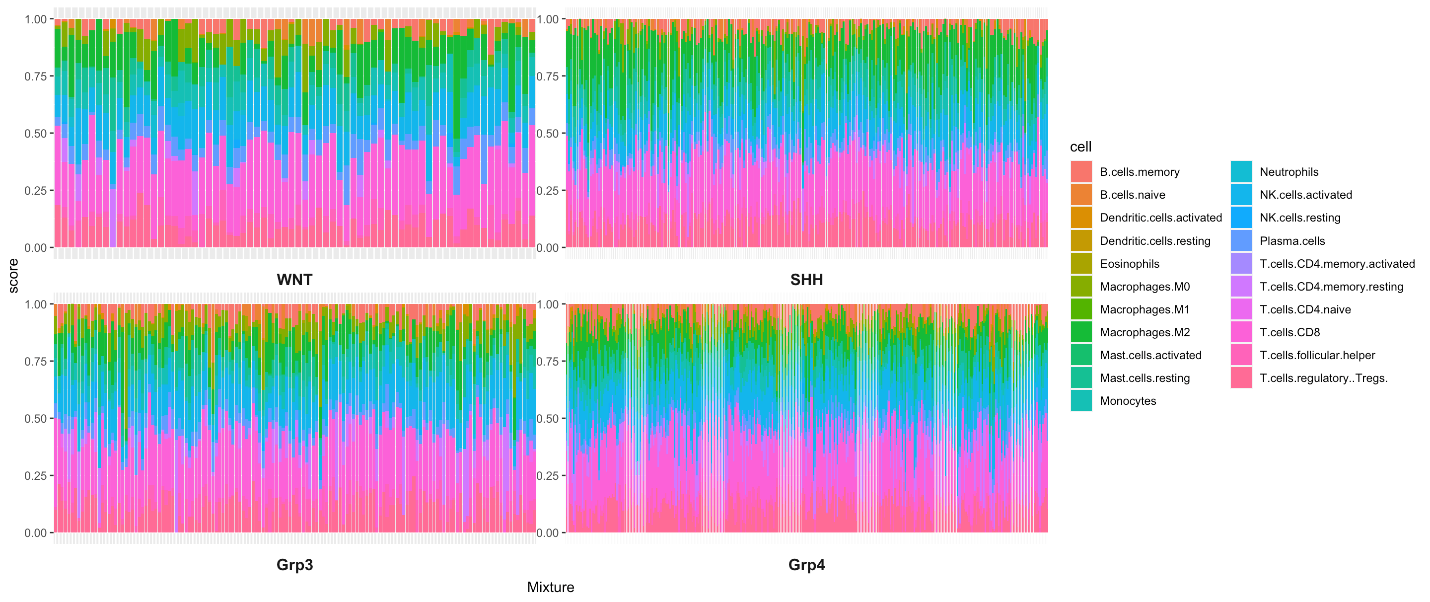


**S5. CIBERSORT for cell fractions in pediatric brain tumor samples.**

Gene expression data from various cohorts was input into CIBERSORTx (https://cibersortx.stanford.edu) where the abundance of various cell populations was estimated. (A) DIPG cohort, (B) ATRT cohort, (C) Medulloblastoma cohort, Swartling, and (D) Medulloblastoma cohort, Cavalli.

**Supplemental Figure 6:**


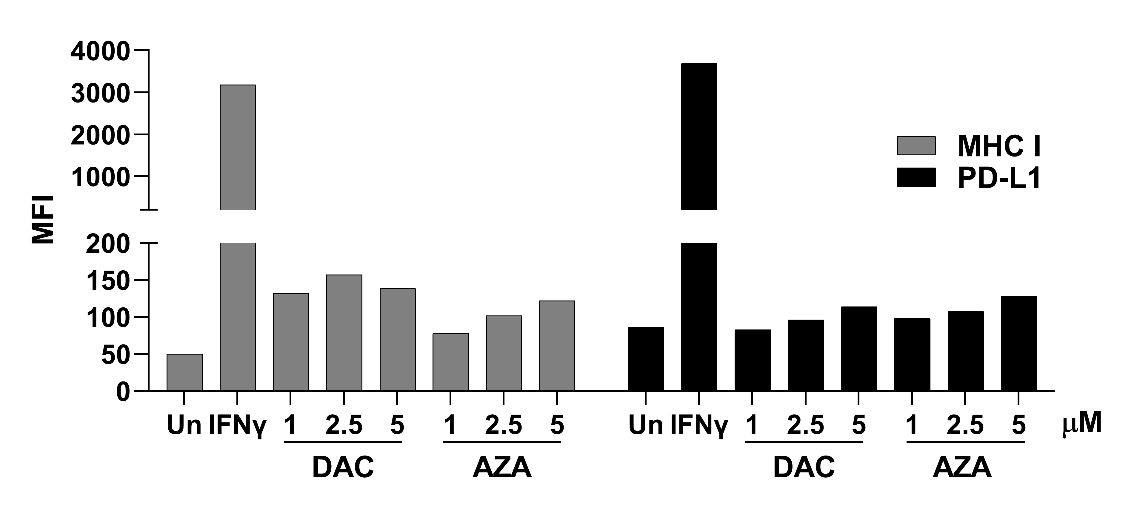
**A.**


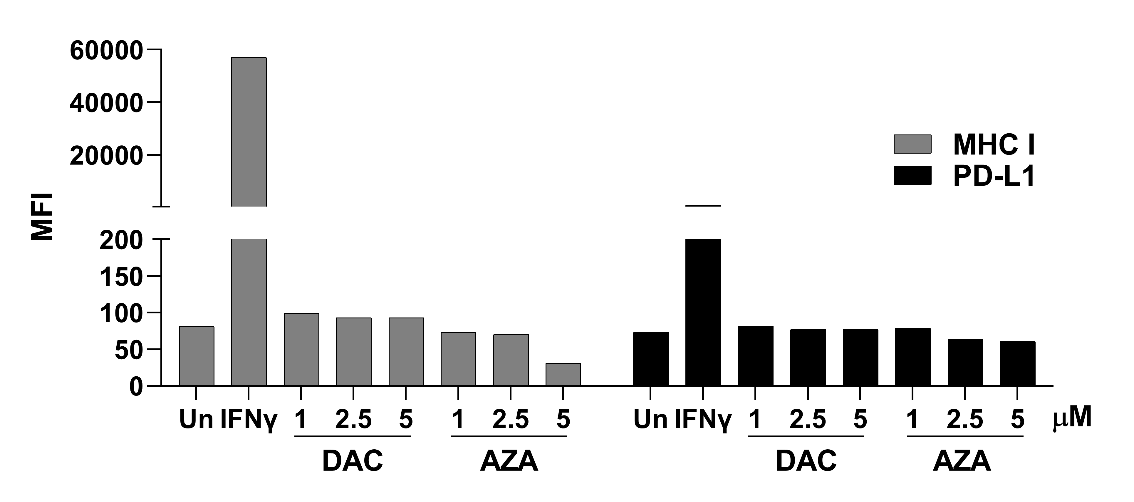
**B.**

**S6. DAC and AZA treatment of additional PBT cell lines.**

Induction of MHC I and PD-L1 expression measured by flow cytometry in (A) BT-16 ATRT cells and (B) Med-2112FHTC medulloblastoma cells following a 2-day treatment with DAC or AZA. Un = Untreated. IFNγ (50 ng/ml for 48 hours) was used as a positive control.

**Supplemental Figure 7:**

**S7. Treatment of IUE-24-C5 GEMM line with DAC for 7 days.**

IUE-24-DIPG cells were treated with different concentrations (25nM, 50nM, 100nM, 250nM, 500nM) of decitabine for 7 days . Viability and total cell counts were determined (n=2).

**Supplemental Figure 8:**

**S8. DNA methyltransferase inhibitor DAC induces MHC I expression in mouse syngeneic brain tumor cell lines.**

Baseline expression of MHC I and PD-L1 in IUE-24-C5, SHH57835, and 7444 mouse brain tumor cell lines (Untreated). Induction of MHC I expression following 2- or 4-day treatment of 0.5 μM DAC. IFNγ (50 ng/ml for 48 hours) was used as a positive control. MFIR equals the median fluorescence intensity of stained cells divided by the isotype control. Horizontal line indicates an MFIR of 1.

**Supplemental Figure 9:**

**­­­**


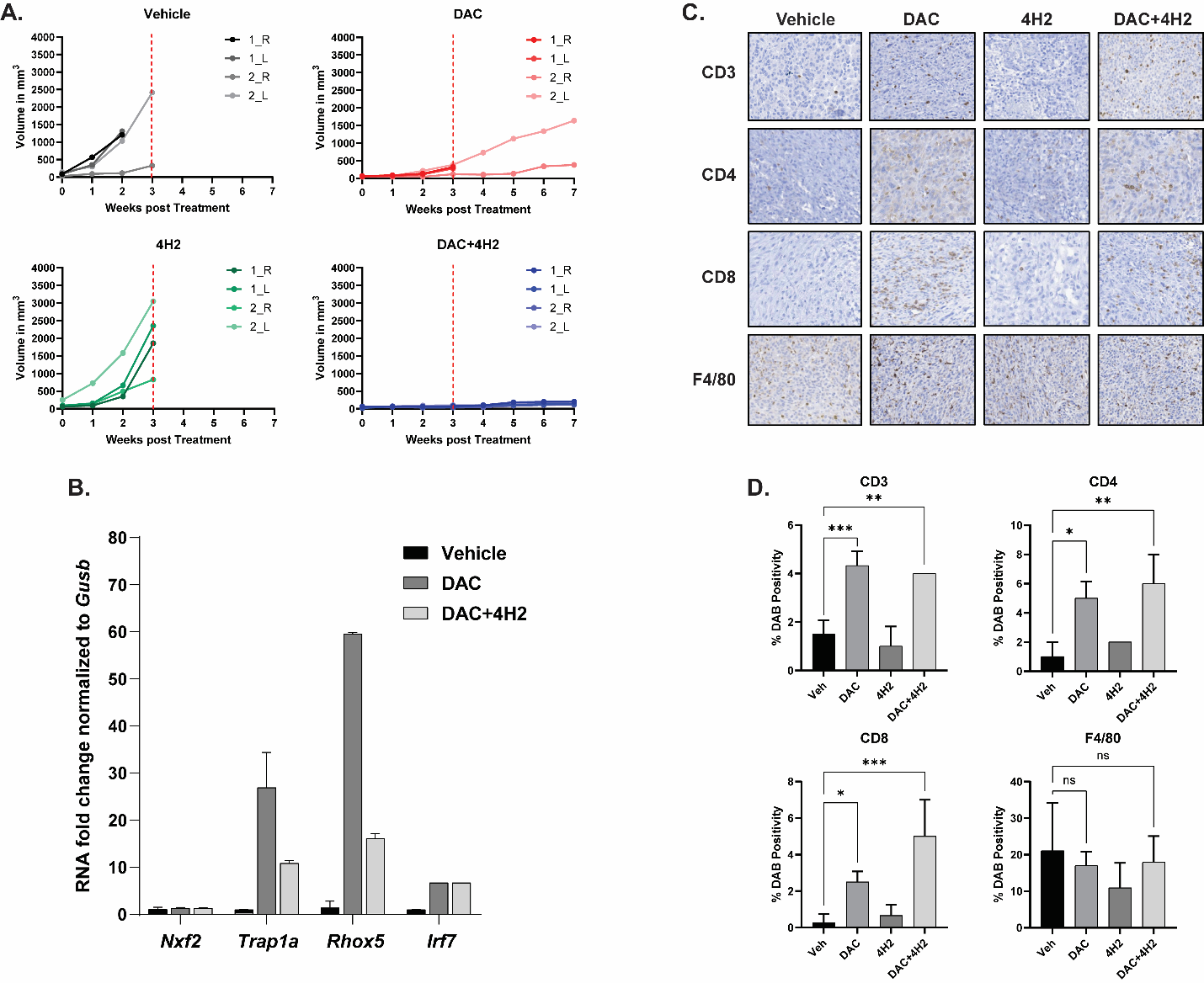


**S9. DAC in combination with 4H2 showed reduced tumor growth in the IUE-24-C5 syngeneic flank model.**

(A) Tumor cells were implanted in both right and left flanks of C57BL/6J mice and tumor volume was calculated by caliper measurements weekly. Changes in tumor growth were analyzed after 3 weeks of treatment (vertical red hatched line). DAC and DAC+4H2 treatment showed significantly smaller tumors compared to vehicle and 4H2 treatment alone. (B) Gene expression analysis of flank tumors demonstrated that DAC treatment alone or in combination with 4H2 can induce *Trap1a* and *Rhox5* neoantigen expression along with *Irf7*, a transcription factor that regulates the expression of type I interferon genes. (C) IHC to identify the presence of various immune cell infiltrates in the flank tumors. (D) Quantitation of immune cell infiltrates as determined by percent DAB positivity.

**Supplemental Figure 10:**


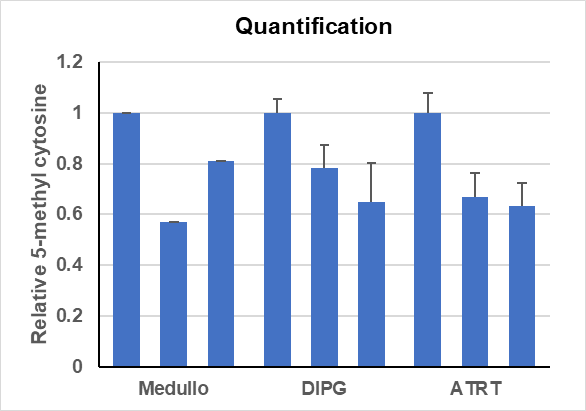


Vehicle

DAC

DAC+4H2

Vehicle

DAC

DAC+4H2

Vehicle

DAC

DAC+4H2

**S10. 5-methylcytosine-specific dot blot assay.**

A 5-methylcytosine-specific dot blot assay was used to evaluate activity of DAC in mouse brain tumor tissue. Density of dot blots were measured and plotted as bar graphs.

**Supplemental Figure 11:**


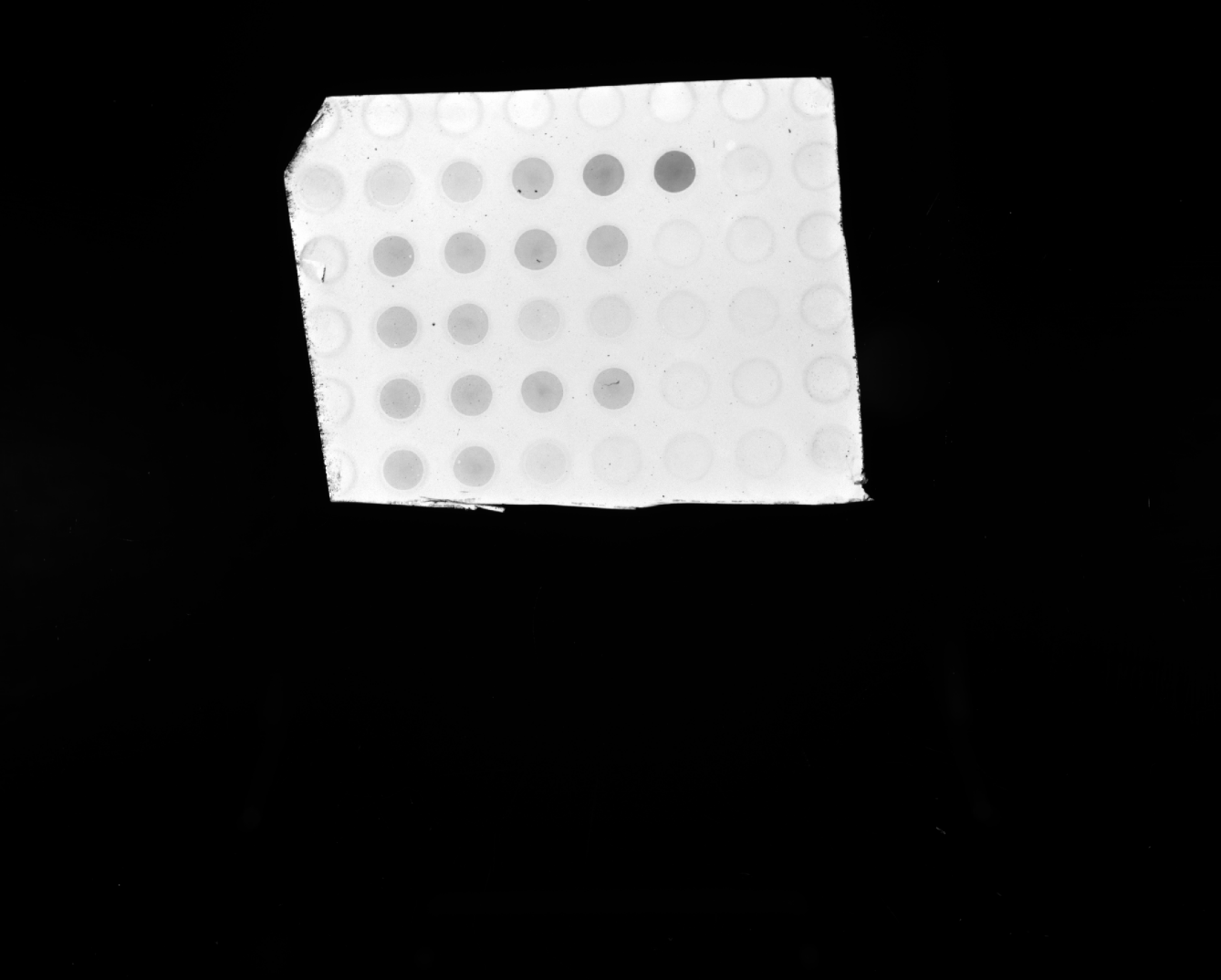


**CTRL**

**DAC**

**4H2**

**DAC+4H2**

**CTRL**

**DAC**

**4H2**

**DAC+4H2**

**IUE-24-C5**


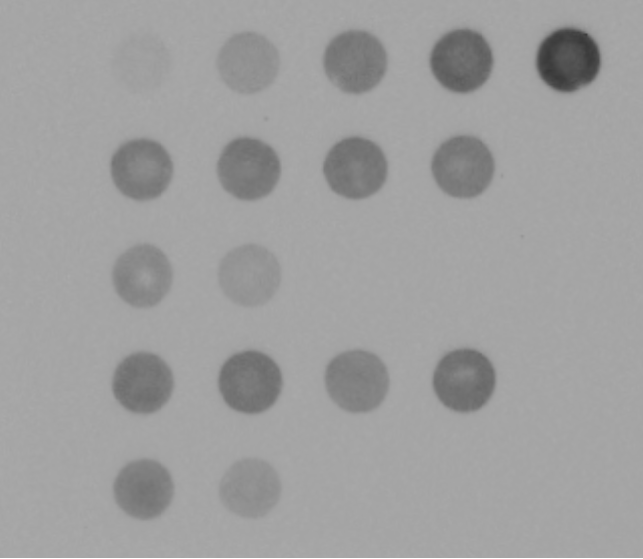


**Methylene blue**

**S11. 5-methylcytosine-specific dot blot assay.**

Genomic DNA (gDNA) was isolated from IUE-24-C5 DIPG tumor model and subjected to treatment with DAC, 4H2, or a combination of both. Each dot contained 100 ng of gDNA, and methylene blue staining was utilized as a loading control.

**Supplemental Figure 12:**

**
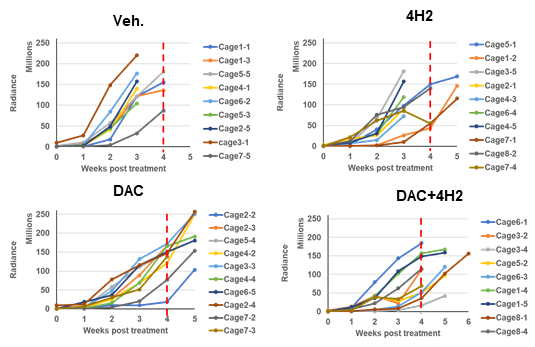
**

**A.**

**
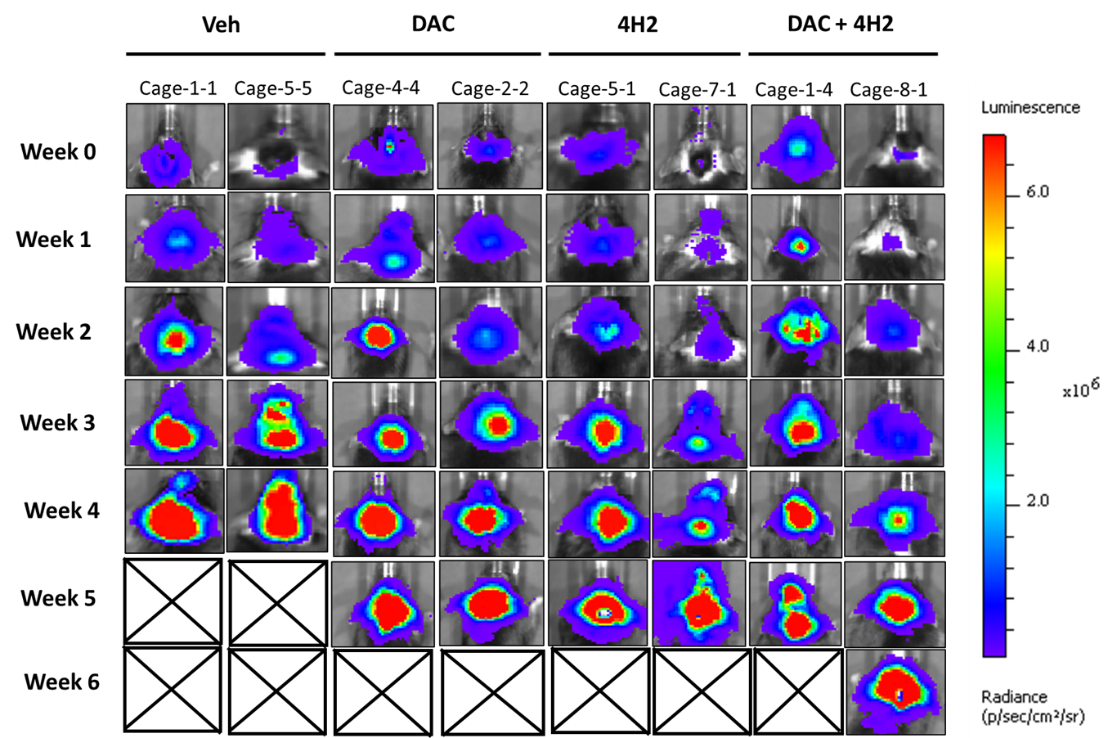
**

**B.**

**S12. DAC and its combination with 4H2 showed improved survival in DIPG orthotopic model.** (A) Luminescence of tumors over time as determined by IVIS imaging. DAC and DAC+4H2 treatment groups showed significantly improved survival (p<0.0001 and 0.0170, respectively compared to vehicle). Red vertical hatched line indicates 4-week post treatment commencement. (B) Representative IVIS images of mice from each treatment group.

**
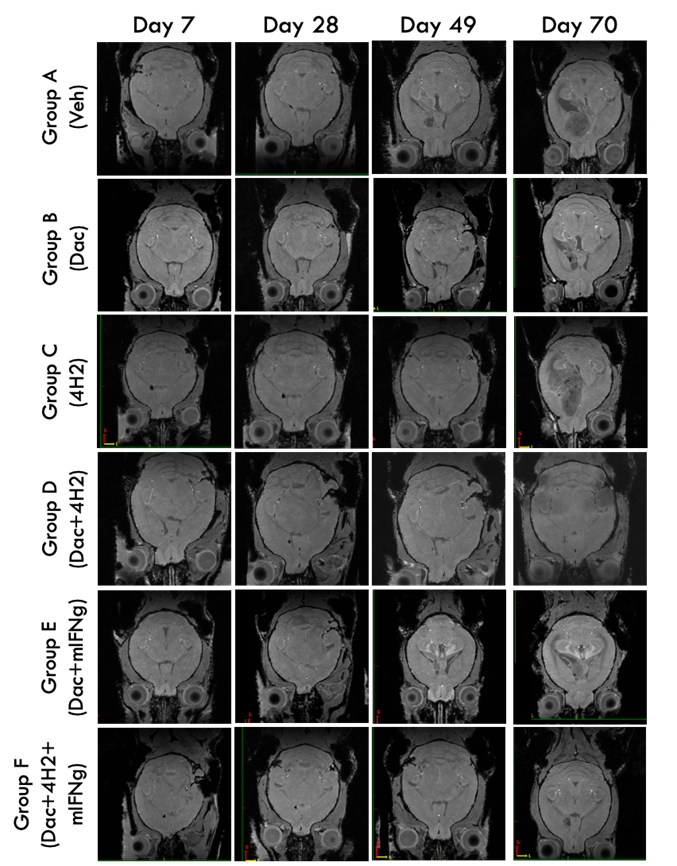
Supplemental Figure 13:**


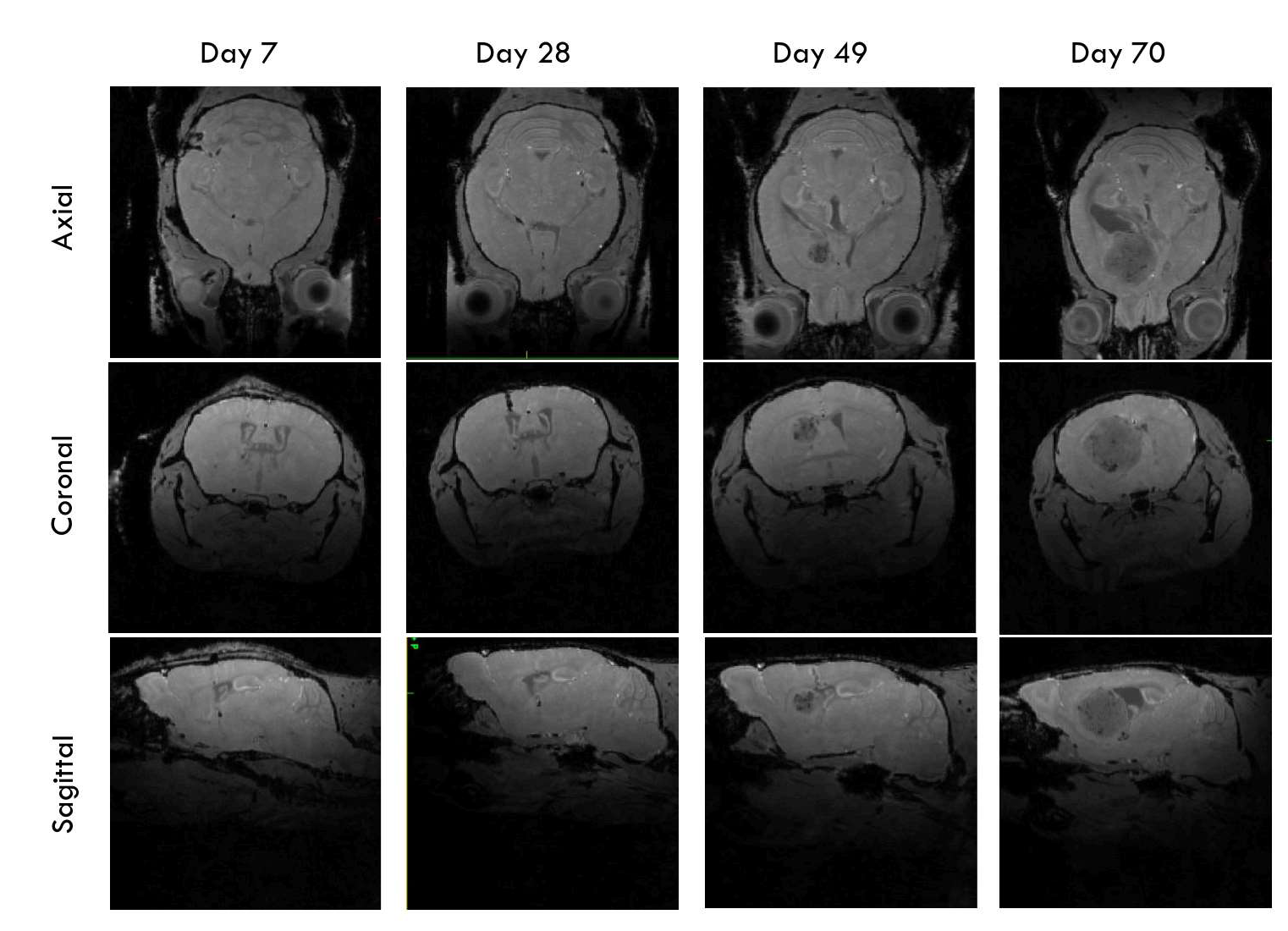


Group A

Group B

Group C

Group D

**S13. MRI monitoring of ATRT tumors.** Representative scans (axial, coronal, sagittal) taken at various time points during the study.

**Supplemental Figure 14:**

**A.**

**B.**

**S14. 7444 model survival curve and neoantigen expression in tumor tissue.**

(A) Kaplan-Meier curves representing survival data for 7444 medulloblastoma model. (B) Evaluation of *Trap1a* neoantigen gene expression using quantitative PCR in tumor tissue harvested from 7444 syngeneic mice treated with either DAC, 4H2, or DAC+4H2.

**Supplemental Figure 15:**


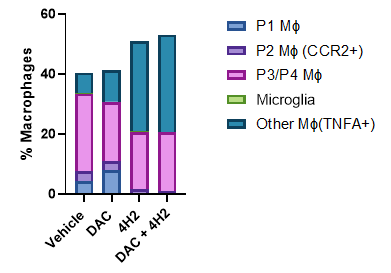

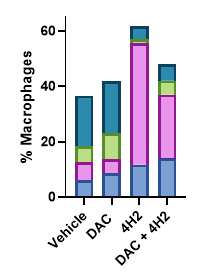

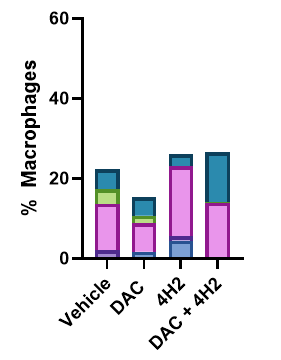

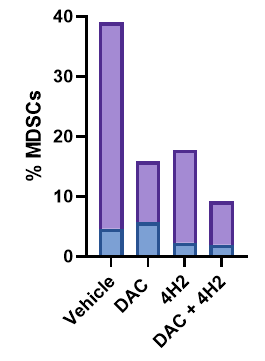

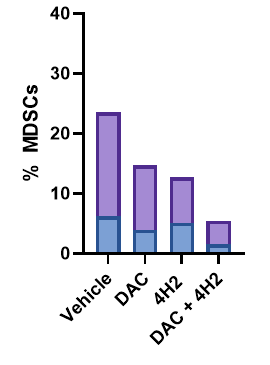

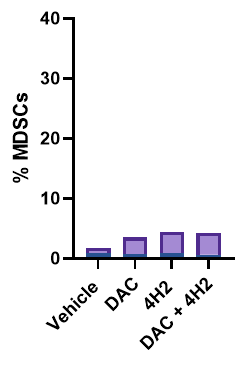


1. **IUE-24-C5 (DIPG)**

**B. IUE-K27M-APP (DMG)**

**C. SU2C_54_i_5 (ATRT)**


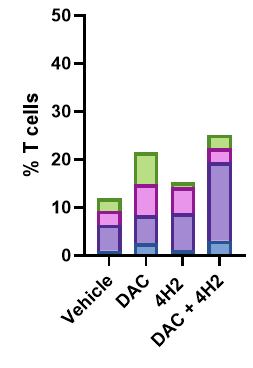

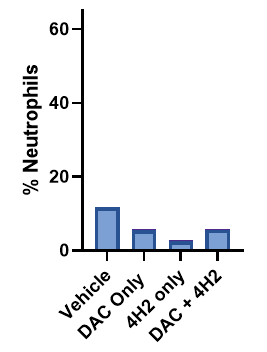

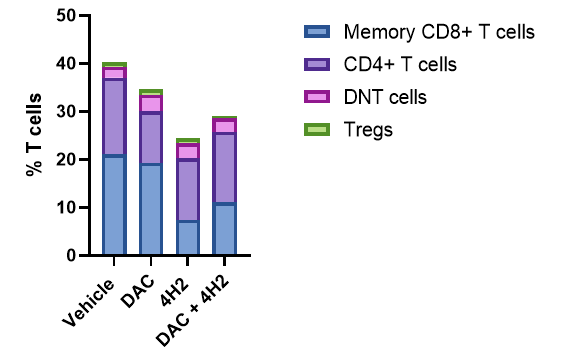

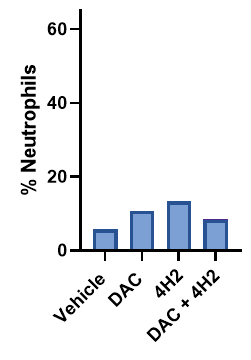

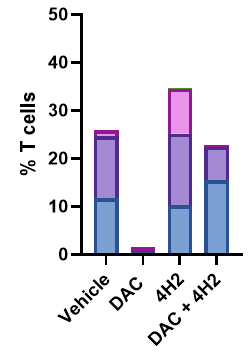

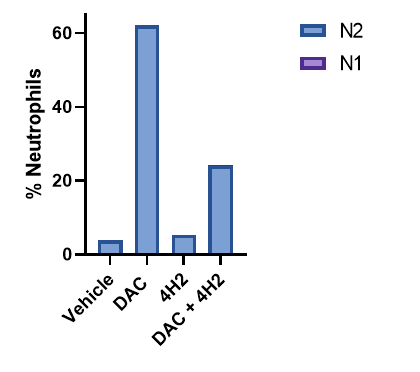

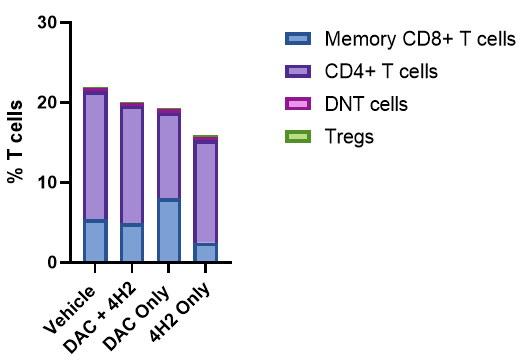

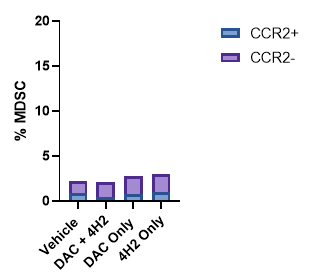

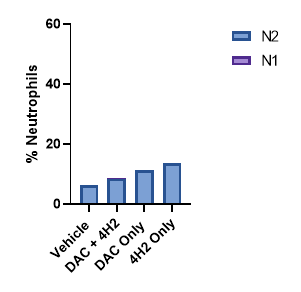

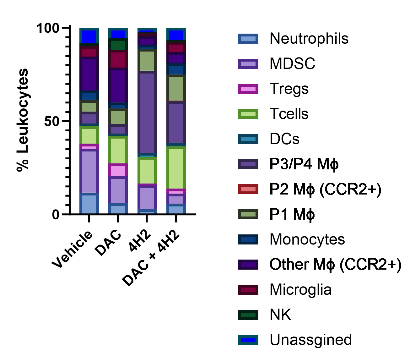

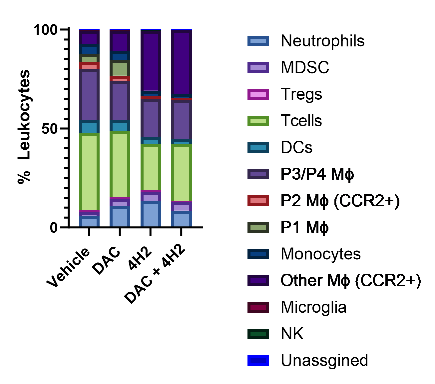

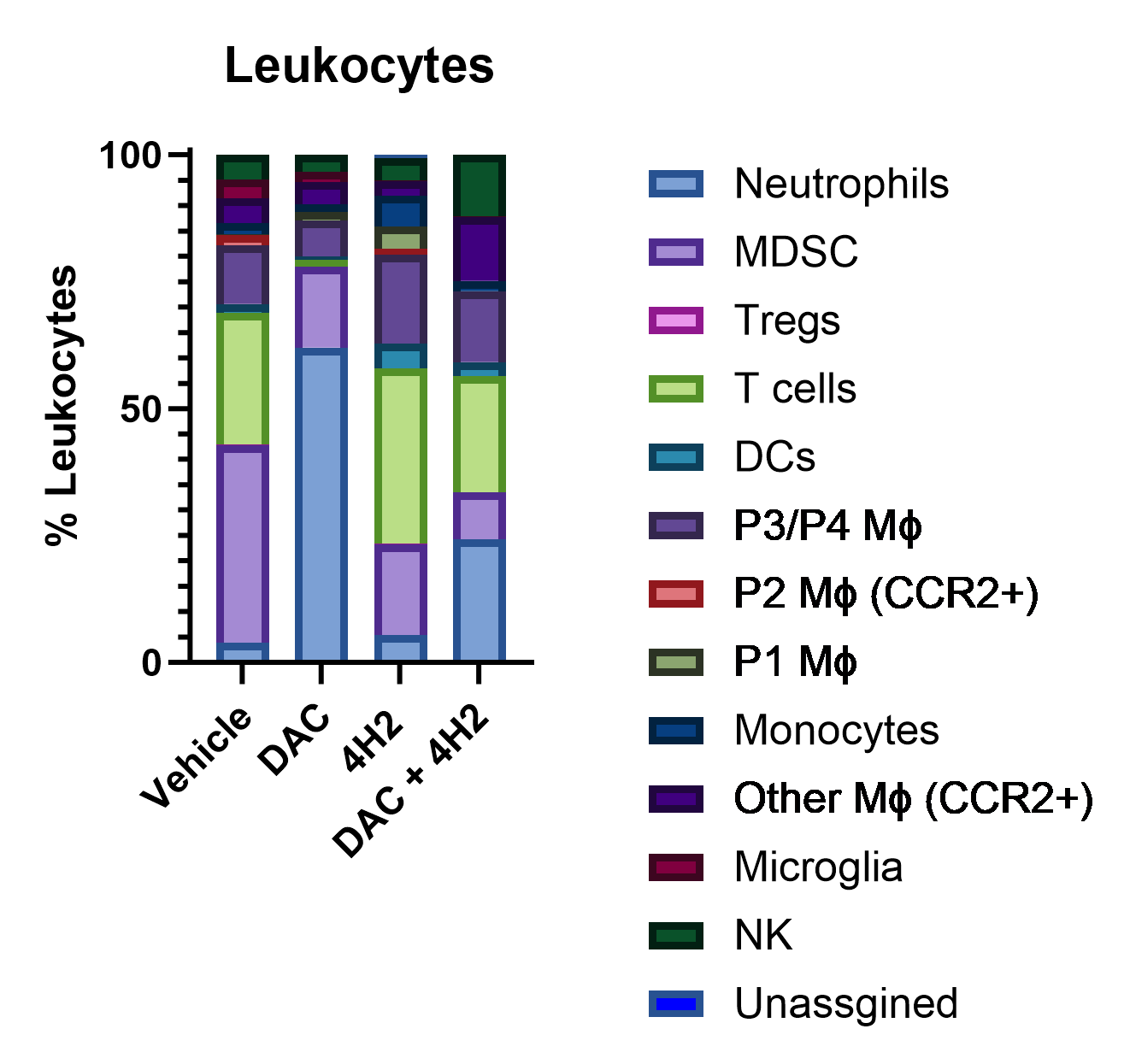


­

**S15. Additional CyTOF data.**

Relative percentage of leukocytes, macrophages, MDSCs, neutrophils, and T cells in brain tumor tissue collected from vehicle, DAC, 4H2, and DAC+4H2 treated mice.

**Supplemental Figure 16:**

**A.**

**B.**

**S16. T cell infiltration.**

DIPG and ATRT models with different treatments were assessed using CyTOF after tissue dissociation. (A) T cell populations were analyzed in mice injected with IUE-24-C5. (B) T cells were analyzed in mice injected with ATRT tumor cells.
